# *Sergey* gen. n., a new doryctine genus from temperate forests of Mexico and Cuba (Hymenoptera, Braconidae)

**DOI:** 10.3897/zookeys.589.8291

**Published:** 2016-05-16

**Authors:** Juan José Martínez, Rubi Nelsi Meza Lázaro, Carlos Pedraza-Lara, Alejandro Zaldívar-Riverón

**Affiliations:** 1 CONICET-Departamento de Ciencias Biológicas, Facultad de Ciencias Exactas y Naturales, Universidad Nacional de La Pampa, Santa Rosa, La Pampa, Argentina; 2 Colección Nacional de Insectos, Instituto de Biología, Universidad Nacional Autónoma de México, Ciudad de México, México; 3 Licenciatura en Ciencia Forense, Facultad de Medicina, Universidad Nacional Autónoma de México, Ciudad de México, México

**Keywords:** Doryctinae, Ichneumonoidea, incomplete lineage sorting, new species, taxonomy

## Abstract

The new doryctine genus *Sergey* **gen. n.** is described with four new species (*Sergey cubaensis* Zaldívar-Riverón & Martínez, **sp. n.**, *Sergey coahuilensis* Zaldívar-Riverón & Martínez, **sp. n.**, *Sergey tzeltal* Martínez & Zalídivar-Riverón, **sp. n.**, *Sergey tzotzil* Martínez & Zalídivar-Riverón, **sp. n.**) from temperate forests of Mexico and Cuba. Similar to many other doryctine taxa, the new genus has a considerably elongated, petiolate basal sternal plate of the first metasomal tergite, although it can be distinguished from these by having the mesoscutum sharply declivous anteriorly with sharp anterolateral edges. The described species have been characterised molecularly based on two mitochondrial (COI, cyt *b*) and one nuclear (28S) gene markers. Based on the mitochondrial gene genealogies reconstructed, the evidence suggests the existence of incomplete lineage sorting or hybridization in the populations from Chiapas and Oaxaca assigned to *Sergey tzeltal* **sp. n**.

## Introduction

The braconid wasp subfamily Doryctinae is a highly diverse, cosmopolitan group that currently comprises 198 genera and about 1,700 species (Braet 2016; Yu et al. 2012). This group gathers a wide array of genera with distinct morphologies and biologies, though most of its species appear to be idiobiont ectoparasitoids of bark-boring or xylophagous beetle larvae (Belokobylskij 1992; Marsh 1997). Previous attempts trying to elucidate the phylogenetic relationships among doryctine genera based on morphological evidence yielded poorly resolved hypotheses (Belokobylskij 1993; Belokobylskij et al. 2004). Subsequent molecular phylogenetic studies carried out for the subfamily (Zaldívar-Riverón et al. 2007, 2008) refuted most of the previously proposed tribes and subtribes (Belokobylskij 1993; Fischer 1981). These molecular phylogenies have served as a base to start building a stable higher-level classification for the group (Zaldívar-Riverón et al. 2007, 2008, 2014; Samacá-Sáenz et al. 2016).

One of the main external morphological features that was traditionally used to group genera within the Doryctinae is the relative length of the basal sternal plate of the first metasomal tergite (acrosternite *sensu* Belokobylskij 1995). This structure can be petiolate, tubular and long or sessile and short (Belokobylskij 1995; Marsh 1997). Within the Doryctinae, a long and tubular basal sternal plate has been shown to have independently evolved in various unrelated genera. Two of these genera are among the most speciose within the subfamily, the cosmopolitan, mainly Old World *Spathius* Nees, and the exclusively Neotropical *Notiospathius* Mathews & Marsh.

In a recent molecular phylogenetic study of *Notiospathius*, various species originally assigned to this genus were nested in two distantly related clades (Ceccarelli and Zaldívar-Riverón 2013). Members of these two clades have consistent external morphological features that distinguish them from each other and from the remaining doryctine genera. Species of one of these clades were placed in the newly described genus *Bolivar* Zaldívar-Riverón & Rodríguez-Jimenez (Zaldívar-Riverón et al. 2013).

In this work, a new doryctine genus, *Sergey* gen. n., is erected to include the species of the second clade, and four new species are described. Three of these species were collected in cloud forests from México and Cuba, whereas the remaining one was collected in a submontane forest in Coahuila, northeast Mexico. Members of the new genus are morphologically distinct from other doryctine genera with petiolate first metasomal tergite by having the anterolateral corners of mesoscutum sharply pointed and a different pattern of ornamentation in the propodeum, with two divergent carinae that sometimes enclose a more or less distinguishable areola. The phylogenetic relationships within the new genus have been assessed based on separate analyses of one nuclear and two mitochondrial (mt) markers, and provide evidence that suggests the existence of incomplete lineage sorting between two populations of one of the described species.

## Material and methods

### Specimens and terminology

Specimens were collected in four different localities in Mexico and Cuba, preserved in 100% ethanol, kept at 20 °C until they were processed for DNA sequencing, and subsequently dried, labelled and mounted. The examined specimens are deposited in the Colección Nacional de Insectos, Instituto de Biología, Universidad Nacional Autónoma de México
(IB-UNAM), Mexico City, Mexico, and the Museo Argentino de Ciencias Naturales “Bernardino Rivadavia” (MACN), Buenos Aires, Argentina.

The morphological terminology follows Sharkey and Wharton (1997), except for the sculpture characters, which follow Harris (1979), and the term precoxal sulcus, which replaces the term sternaulus according to Wharton (2006). Digital colour images were taken with a Leica® Z16 APO-A stereoscopic microscope, a Leica® DFC295/DFC290 HD camera, and the Leica Application Suite® program. Digital SEM images were taken with a FEI® INSPECT (Oregon, USA) and a Hitachi® SU1510 SEM microscopes in low vacuum at the Museo Nacional de Ciencias Naturales
(CSIC, Madrid, Spain) and the IB-UNAM, respectively.

### Gene genealogies

Sequences of three gene markers have been examined for specimens belonging to the new genus. These included 34 and 26 sequences that were previously published in Ceccarelli and Zaldívar-Riverón’s (2013) phylogenetic study of *Notiospathius* belonging to the cytochrome oxidase I (COI; 531 bp) mt and the second and third domain regions of the 28S nuclear ribosomal (r) (~617 bp;) DNA genes, respectively. Moreover, COI and 28S sequences were generated of additional specimens of this genus, as well as sequences of a 371 bp fragment of the cytochrome *b* mt DNA gene for a subset of the examined specimens. Sequences of *Heterospilus tauricus* Telenga (DNA voucher number CNIN884; GenBank accession nos. KC822008, 36, 72 for COI, 28S and cyt *b*, respectively) were also included to root the trees. *Heterospilus* was closely related to the newly described genus in the above molecular phylogenetic study. The sequenced ingroup specimens, their localities and GenBank accession numbers are provided in the description section.

Corrected pairwise genetic distances for the three gene markers were calculated using the K2P model with MEGA version 6 (Kimura 1980; Tamura et al. 2013). Separate gene genealogies were carried out with the program MrBayes version 3.2.6 (Ronquist et al. 2012) in the Cipres Science Gateway (Miller et al. 2010). Each analysis consisted of two independent runs of 20 million generations each, used uniform priors and sampled trees every 1000 generations. The following evolutionary models selected for each partition were obtained using the Bayesian criterion with JMODELTEST2 (Darriba et al. 2012): 28S.- K2; CytB.- 1^st^ pos, TrN + G, 2^nd^ pos, HKY + I, 3^rd^ pos, GTR + G; COI.- 1^st^ pos, F81, 2^nd^ pos F81, 3^rd^ pos HKY. Burn-in was determined assessing convergence between runs verifying the potential scale reduction factors (PSRF) and the estimated sample size (ESS) for all tested parameters. The burn-in fraction was set to 0.25, which corresponded to 5,000 trees (5 × 10^6^ generations) in all analyses. The remaining trees from the two independent runs were employed to reconstruct a majority rule consensus tree using the ‘halfcompat’ option implemented in MrBayes. Clades were regarded as significantly supported if they had a posterior probability 0.95 (Ronquist et al. 2012).

## Results and discussion

### Sergey gen. n.

Taxon classificationAnimaliaHymenopteraBraconidae

http://zoobank.org/0C1D768E-779F-42A9-BC6A-520BA2447E06

Figs 1–2

#### Diagnosis.

Species of this new genus can be distinguished from members of the remaining doryctine genera with long, petiolate first metasomal tergite (*e.g. Bolivar*, *Notiospathius*, *Pecnobracon* Kieffer et Jöergensen, *Spathius*, *Trigonophasmus* Enderlein) by having the mesoscutum sharply declivous anteriorly with sharp anterolateral corners. *Sergey* could be included in the key to dorcytine genera of the New World (Marsh 1997) as follows:

**Table:** 

69 (65)	First subdiscal cell of fore wing open at apex, 2cu-a absent, occasionally an infuscate spot or short line present between 2–1A and 2CUa but no distinct vein present (Fig. 28)	**70**
-	First subdiscal cell closed at apex, 2cu-a present and distinctly meeting 2–1A (Fig. 30)	**75**
70 (69)	Anterolateral corners of mesoscutum sharply pointed into two flanges, metasoma petiolated	***Sergey* gen. n.**
-	Anterolateral corners of mesoscutum not sharply pointed anteriorly, the mesoscutum may be sharply raised anteriorly with respect to the pronotum, but flanges are not present, metasoma variable	**70**’
70’(70)	Most of mesosoma smooth and shining, mesonotum occasionally coriaceous, propodeum occasionally rugose	**71**
-	Most of mesosoma sculptured, rugose or coriaceous, at most mesopleuron smooth below sternaulus (precoxal sulcus)	**73**

#### Description.

*Head*: not depressed. Ocelli arranged in almost equilateral triangle. Frons not distinctly excavated, without a median keel between antennal sockets. Occipital carina complete, fused with hypostomal carina before mandible. Malar suture absent. Clypeus not high, delineated from face by distinct furrow, with fine lower flange. Hypoclypeal depression wide, round. Postgenal bridge narrow. Maxillary palpi 5-segmented, apical segment longer than fourth segment; labial palpi short, 4-segmented, third segment not shortened. Scape of antenna wide and rather short, without flange apically and ventroapical lobe, without basal constriction; ventral margin of scape shorter than dorsal margin in lateral view. First flagellar segment about the same length as second segment, usually several apical or subapical segments whithish. Apical segment more or less pointed apically, without “spine”.

**Figures 1–2.**
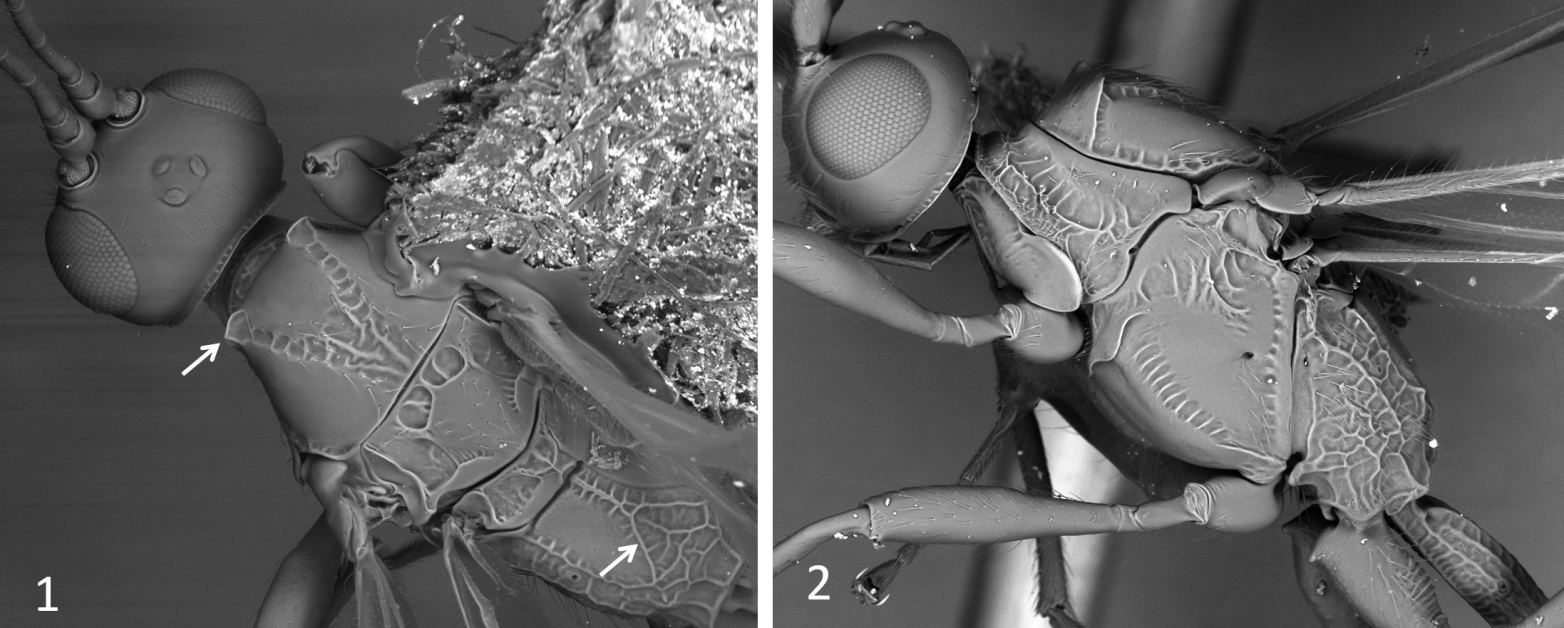
SEM images of *Sergey tzeltal* sp. n.; **1** head and mesosoma in dorsal view **2** head and mesosoma in lateral view. Arrows indicate anterolateral sharp edges/flanges on mesoscutum and divergent carinae on propodeum.

*Mesosoma*: not depressed. Neck of prothorax short but visible in dorsal view. Pronotum dorsally weakly convex (lateral view), with a transverse carina and a scrobiculate pronotal sulcus. Pronope absent. Propleural dorsoposterior flange rather short. Mesonotum distinctly elevated above pronotum. Anterolateral corners of mesoscutum projected in two flanges (Figs 1–2). Notauli present and complete, scrobiculate, obscured in rugose median area of mesoscutum. Scuto-scutellar (transscutal) suture distinct and complete. Prescutellar depression, with 3–5 high carinae. Scutellum slightly convex, subtriangular in dorsal view, about as long as wide, without lateral carinae. Subalar depression distinct. Mesopleuron with subalar sulcus, sternaulus and posterior mesopleural sulcus coarsely sculptured, otherwise smooth and polished. Mesopleural pit distinct. Precoxal sulcus (sternaulus) rather deep, wide, and scrobiculate, extended at least two thirds length of mesopleuron. Prepectal carina distinct and complete, laterally reaching anterior margin of subalar depression. Propodeum with two dorsolateral areas delimited by distinct carinae; sometimes these divergent carinae suggest an areola enclosing a rugose area (Fig. 1), in other cases the propodeum is uniformly rugose-areolate beyond the dorsolateral areas. Propodeal bridge absent. Propodeal spiracles small and round. Metapleuron slightly convex, entirely sculptured, rugose-areolate.

*Wings*: veins RS and r-m present, thus first and second submarginal cells entirely closed. Second submarginal cell rather long and narrow. First subdiscal cell open postero-apically, vein 2cu-a absent. Veins 1a and 2a absent. Hind wing with vein C+Sc+R longer than vein SC+R. Vein RS arising from vein R far from vein r-m. Marginal cell more or less distinctly narrowed towards apex, without vein r. Vein cu-a present. Vein M+CU about 0.6-0.7 times as long as 1M; vein m-cu straight. Male hind wing without stigma-like swelling of basal veins.

*Legs*: Fore tibia on inner surface with several long and slender spines arranged along its anterior margin in almost single vertical line. Hind coxa long and narrow, with basoventral tubercle. Claws simple.

*Metasoma*: first tergite petiolate, long and narrow, usually striate-coriaceous, with some transverse carinae basally, these carinae sometimes reduced. Basal sternal plate (acrosternite) of first tergite long, 0.6–0.7 times as long as first tergite, extended distinctly beyond level of spiracles. Dorsope of first tergite small and shallow; spiracular tubercles indistinct, situated in basal 0.3 of tergite. Second tergite without distinct furrows and areas. Second suture considerably shallow, complete, almost straight in females and distinctly curved in males. Third tergite without transverse furrow and basal area. Tergites behind second with a single transverse line of sparse long erect setae. Ovipositor distinctly darkened apically, with two distinct subapical nodes. Ovipositor sheaths long, about as long as metasoma or slightly longer.

#### Etymology.

We are very pleased to name this genus after our dear friend and colleague Dr. Sergey A. Belokobylskij, for his great contribution to the taxonomic knowledge of the braconid subfamily Doryctinae. Gender is to be considered masculine.

#### Type species.

*Sergey tzeltal* sp. n.

#### Key to species of *Sergey*

**Table:** 

1	Eyes small, their height about as long as malar space (Fig. 4); first metasomal tergite 1.5 times longer than its apical width (Fig. 8) (state of Coahuila, Mexico)	***Sergey coahuilensis* sp. n.**
–	Eyes big, their height distinctly longer than malar space (Figs 21, 31); first metasomal tergite slender, at least two times longer than its apical width	2
2	Head and mesoscutum distinctly sculptured, transversally striate (Figs 13, 17; fore wing with vein m-cu reaching vein RS+M basally to 2RS, thus vein (RS+M)b present and distinct (Fig. 18) (Cuba)	***Sergey cubaensis* sp. n.**
–	Head and mesoscutum mostly smooth and polished (Figs 9, 22, 32, 33); fore wing with vein m-cu reaching vein RS+M, interstitial with respect to vein 2RS, thus vein (RS+M)b absent (Figs 7, 28) (Mexico)	**3**
3	Antenna with a white apical or subapical band composed of 3-7 (rarely two) flagellomeres in females (Figs 24, 26); males either with two apical flagellomeres whithish (Fig. 25), or with antenna entirely brown (Fig. 27) (states of Oaxaca and Chiapas)	***Sergey tzeltal* sp. n.**
–	Antenna of females with a subapical band only composed of the articulation between the 19^th^ and 20^th^ flagellomeres, five apical flagellomeres brown (Fig. 34), males with antenna entirely brown (state of Chiapas)	***Sergey tzotzil* sp. n.**

**Figures 3–8.**
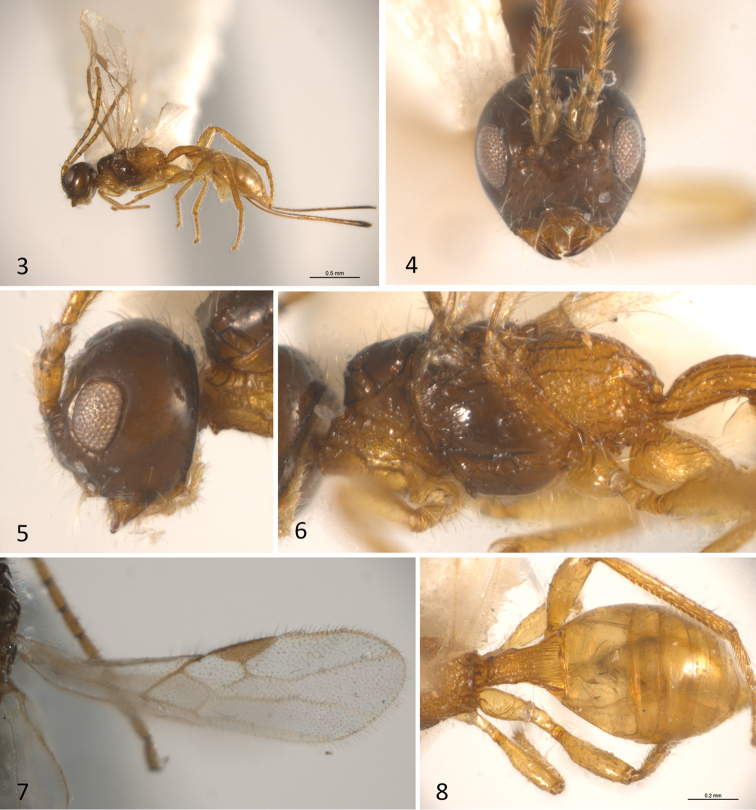
*Sergey coahuilensis* sp. n.; **3** habitus of female in lateral view **4** head in anterior view **5** head in lateral view **6** head and mesosoma in lateral view **7** forewing **8** metasoma in dorsal view.

**Figures 9–10.**
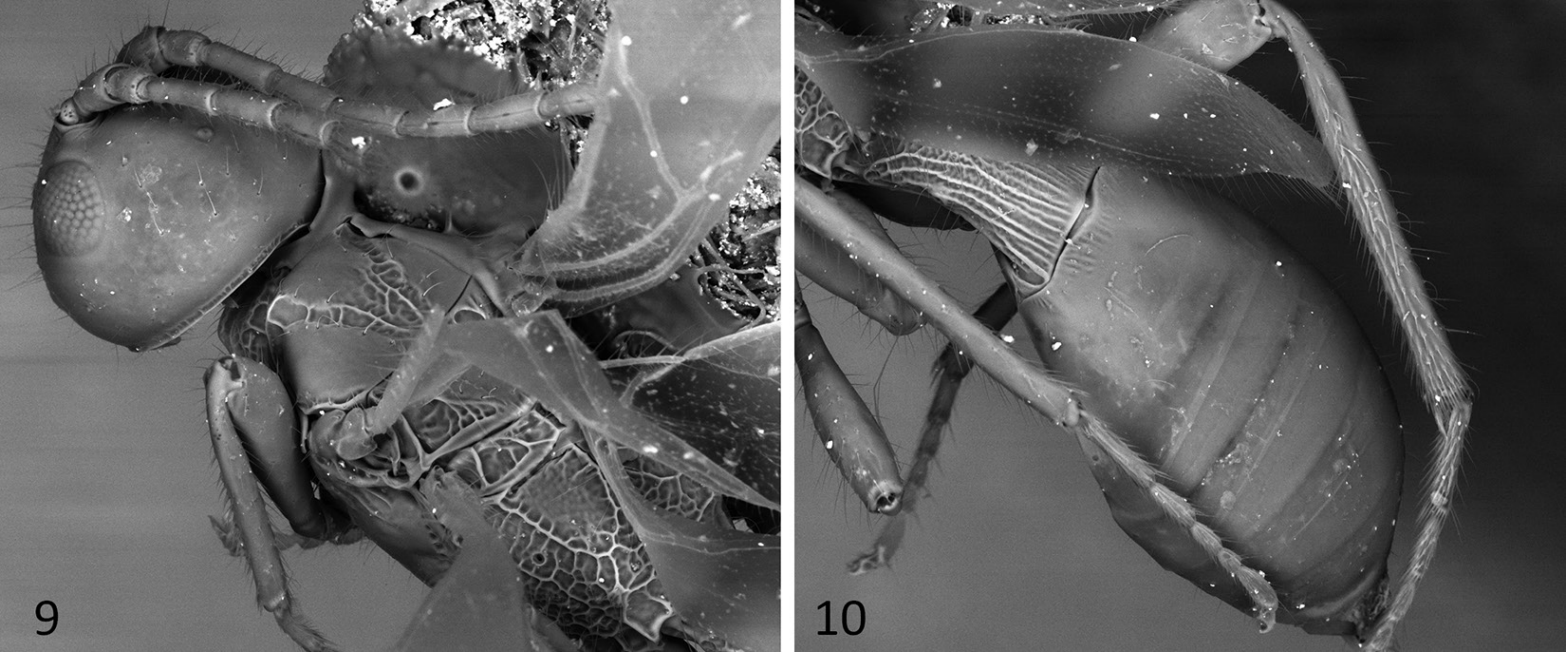
SEM images of *Sergey coahuilensis* sp. n.; **9** head and mesosoma in dorsal view **10** metasoma in dosolateral view.

**Figures 11–17.**
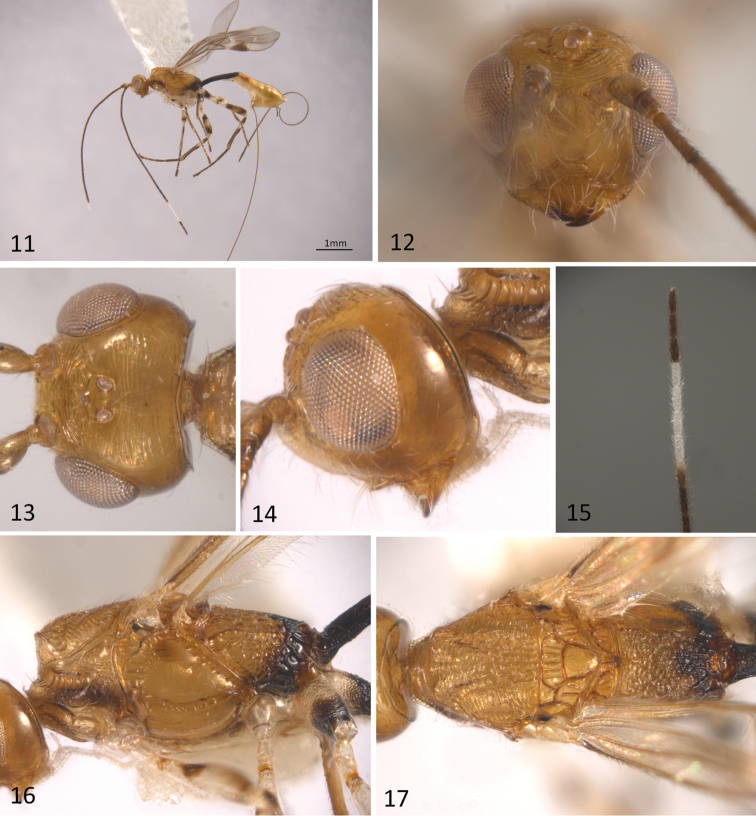
*Sergey cubaensis* sp. n.; **11** habitus of female in lateral view **12** head in anterior view **13** head in dorsal view **14** head in lateral view **15** female apical flagellomeres **16** mesosma in lateral view **17** mesosoma in dorsal view.

**Figures 18–19.**
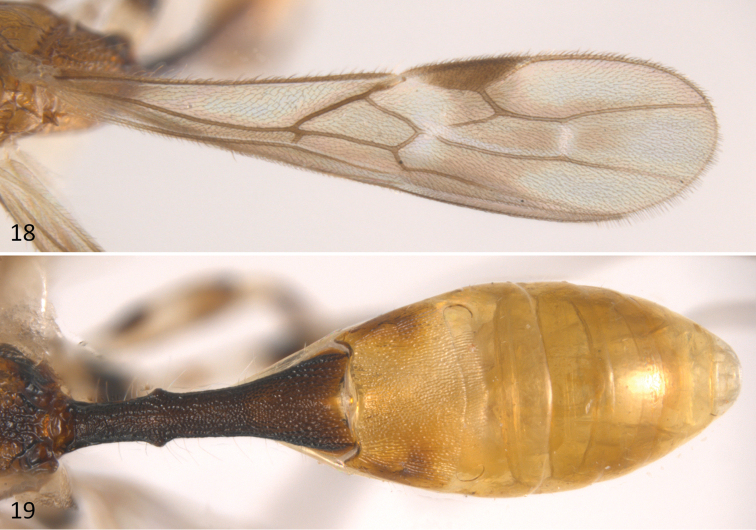
*Sergey cubaensis* sp. n.; **18** fore wing **19** metasoma in dorsal view.

**Figures 20–23.**
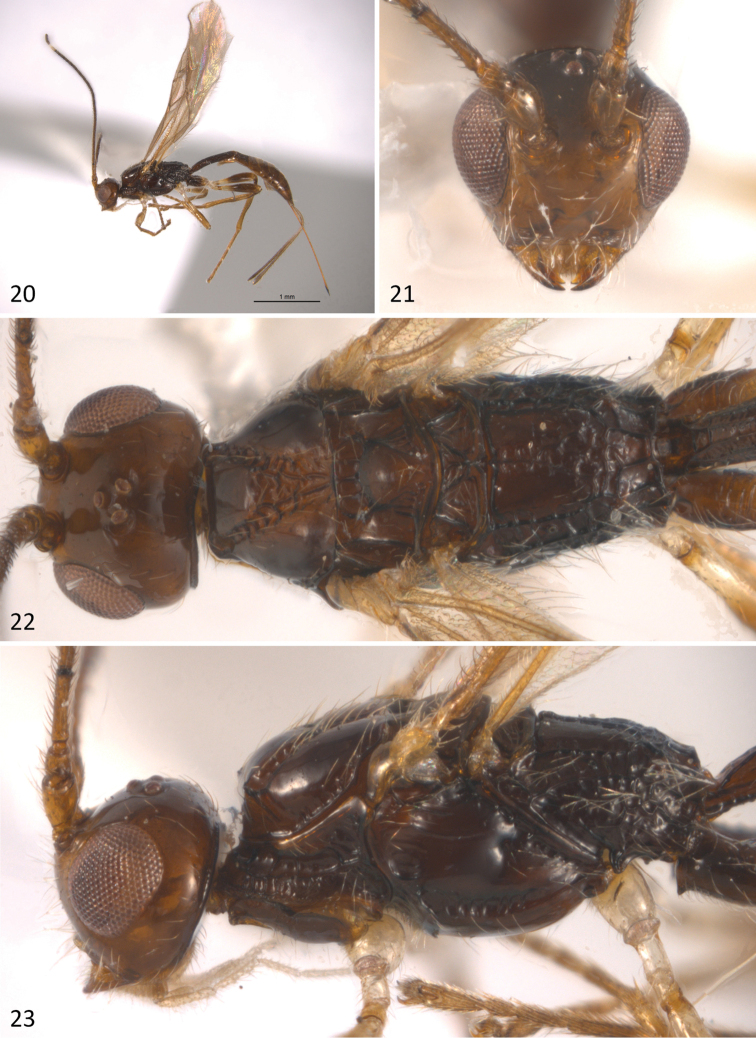
*Sergey tzeltal* sp. n.; **20** habitus of female in lateral view **21** head in anterior view **22** head and mesosoma in dorsal view **23** head an mesosoma in lateral view.

**Figures 24–29.**
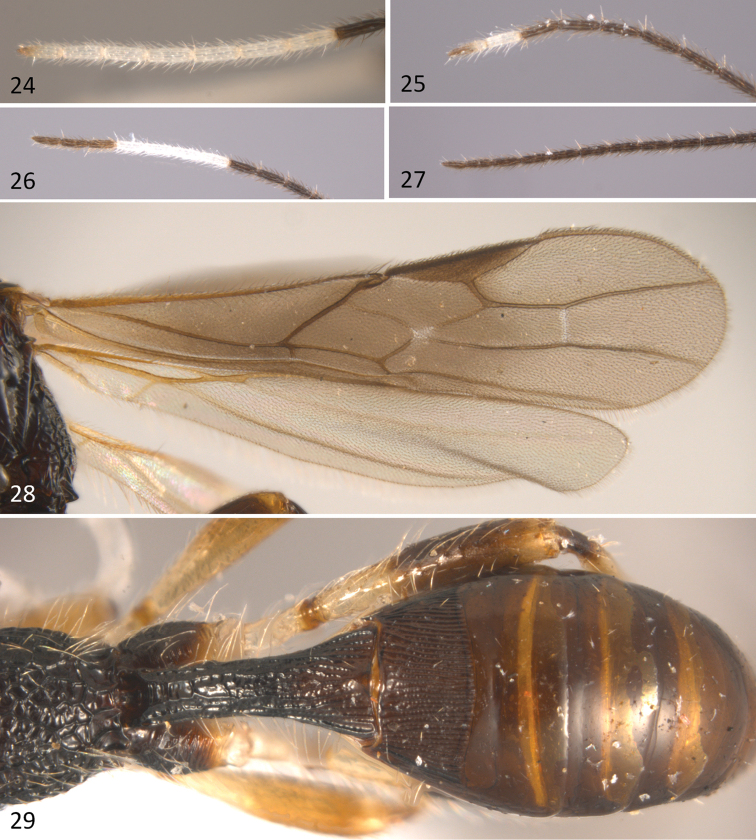
*Sergey tzeltal* sp. n.; **24** antenna of female (Oaxaca) **25** antenna of male (Oaxaca) **26** antenna of female (Chiapas) **27** antenna of males (Chiapas) **28** fore and hind wings **29** metasoma in dorsal view.

**Figures 30–35.**
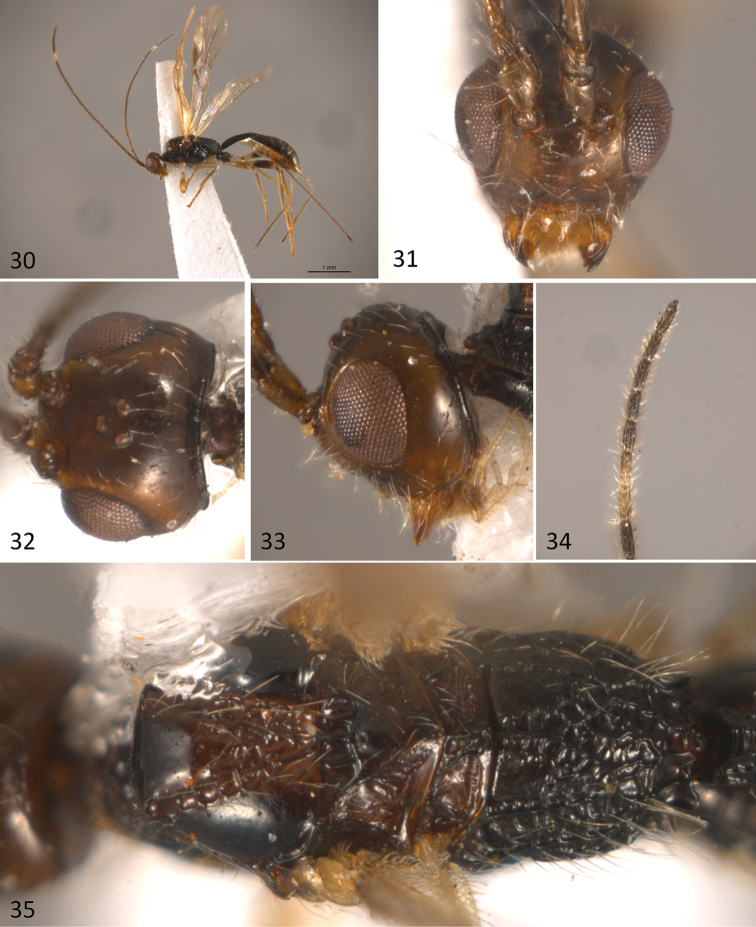
*Sergey tzotzil* sp. n.; **30** habitus of female in dorsal view **31** head in anterior view **32** head in dorsal view **33** head in lateral view **34** female apical flagellomeres **35** mesosoma in dorsal view.

### Sergey coahuilensis

Taxon classificationAnimaliaHymenopteraBraconidae

Zaldívar-Riverón & Martínez sp. n.

http://zoobank.org/A27FD56A-EB2D-42B1-A918-EA9F9CF1AD8A

Figs 3–8 , 9–10

#### Diagnosis.

This is the most distinctive species of the genus. It can be distinguished from the remaining members of *Sergey* by having the eyes considerably smaller, their height about as long as malar space (distinctly longer than malar space in the remaining species); and the first metasomal tergite broad, 1.5 times longer than its apical width (slender, at least 2.1 times longer than its apical width in the remaining species).

#### Description.

Body length 2.1mm (Fig. 3), fore wing 1.5mm; ovipositor sheaths 1.1mm. Colour: head excluding antennae, mesoscutum and mesopleuron brown, otherwise uniformly honey yellow. *Head*: about as high as wide (anterior view) (Fig. 4), 0.7 times as long as wide (dorsal view). Clypeus, face, frons and vertex largely smooth and shining (Fig. 5), with a few shallow rugae near the mandible insertion and antennal sockets; temple smooth. Eye small 1.5 times higher than wide. Malar space height/eye height ratio 1.1 (Fig. 4). Temple/eye length ratio (dorsal view) 0.6. Antenna incomplete, only with nine basal flagellomeres; first flagellomere about four times longer than wide and as long as second.

*Mesosoma*: 2.0 times longer than wide and 1.9 times longer than high (Fig. 6). Pronotal groove wide, and scrobiculate, pronotal carina distinct. Propleuron rugose on median third. Mesoscutum transverse, 0.6 times as long as wide. Mesoscutal lobes smooth, notauli deep and scrobiculate, obscured in an irregularly rugose median area before reaching the scuto-scutellar suture (Fig. 9). Prescutellar sulcus with three carinae, the median one straight, and the lateral ones irregular. Scutellar disc smooth and triangular. Mesopleuron smooth. Precoxal sulcus, deep, wide and scrobiculate-rugose, running along the entire length of mesopleuron. Subalar sulcus deep and rugose. Metanotum with a median carina but without a distinct projection. Metapleuron entirely areolate rugose. Propodeum with two divergent carinae running from median anterior edge delimiting two smooth dorsolateral areas, beyond these carinae it is almost uniformly areolate rugose.

*Wings*: fore wing (Fig. 7) length 3.0 mm, length/width ratio 3.85; vein 1cu-a postfurcal to vein 1M; veins 2RS/2M ratio 0.5.

*Legs*: fore tibia with a row of spines. Hind coxa slightly striate dorsally, smooth ventrally, with a small but distinct basoventral tubercule.

*Metasoma*: basal sternal plate/length of first tergum 0.6. First metasomal tergite 2.8 times longer than apically wide (Figs 8, 10). Second median tergite longitudinally striate on basal one fifth, smooth apically. Suture between second and third median tergites slightly sinuate. Remaining terga smooth and polished. Ovipositor length 1.2mm, 1.1 times as long as metasoma.

**Male**. Unknown.

#### Distribution.

Known only from a submontane forest at the type locality in Coahuila, Mexico.

#### Biology.

Unknown.

#### Etymology.

The specific epithet refers to Coahuila, the Mexican state where the type locality of this species is located.

#### Material examined.

Holotype: female (CNIN), Mexico, Coahuila, Mpio. Torreón, Sierra de Jumillo, Arroyo Palos, 09-11/X/2009, 25.13 N - 103.27 O, 2006 msnm, DNA voucher no. IB-CNIN-637, GenBank accession nos. JN870454 (COI), JN870613 (cyt *b*), KC822013 (EF-1alpha; not included in this work), JN870735 (*wingless*, not included in this work).

### Sergey cubaensis

Taxon classificationAnimaliaHymenopteraBraconidae

Zaldívar-Riverón & Martínez sp. n.

http://zoobank.org/4EB92C3F-9FE0-477A-BB34-A838A602D43B

Figs 11–17 , 18–19

#### Diagnosis.

This distinctive species can be distinguished from the remaining species of *Sergey* by having: 1) a mostly yellow body colour (brown to black in the remaining species); 2) head and mesoscutum distinctly sculptured, transversally striate (entirely smooth and polished in the remaining species); and 3) fore wing with vein m-cu reaching vein RS+M basally to 2RS, thus vein (RS+M)b present and distinct (m-cu reaching vein RS+M interstitial with respect to vein 2RS, thus vein (RS+M)b absent in the remaining species).

#### Description.

Body length 3.1mm (Fig. 11), fore wing 2,5 mm; ovipositor sheaths 3.5 mm. *Colour*: most part of the body yellow; apical third of propodeum and first metasomal tergite dark brown, second metasomal tergite yellow with lateral areas brown; antennae honey yellow, gradually darkening toward apex, subapical 20^th^ to 23^rd^ segments white (Fig. 15), apical three segments dark brown; fore and middle coxae pale yellow; fore and middle tibiae brown to dark brown; trochanter and trochantellus pale yellow; tarsi brown to dark brown; hind coxa pale yellow basally, dark brown apically; hind femur and tibia with four alternate yellow and dark brown transversal bands. Wings hyaline; pterostigma and veins brown. Ovipositor sheaths yellow to honey yellow.

*Head*: 0.7 times as high as wide in anterior view (Fig. 12), 0.6 times as long as wide in dorsal view (Fig. 13). Vertex and frons distinctly striate; face, temple and gena smooth (Fig. 14); clypeus transversally striate. Eye 1.2 times higher than wide. Malar space height/eye height ratio 0.3 (Fig. 12). Temple/eye length ratio (dorsal view) 0.4. Antenna with 26 flagellomeres, first flagellomere about four times longer than wide and as long as second.

*Mesosoma*: about 1.9 times longer than wide and 2.0 times longer than high (Figs 16–17). Pronotal groove wide, deep, and scrobiculate, pronotal carina distinct. Propleuron smooth to slightly rugose. Mesoscutum slightly transverse, 0.7 times as long as wide. Mesoscutal lobes transversally striate with coriaceous microsculpture, notauli deep, complete and scrobiculate (Fig. 17), not joining, reaching the end of mesoscutum, obscuring in an irregular longitudinal rugose median area before reaching the scuto-scutellar suture. Prescutellar sulcus with four distinct carinae. Scutellar disc smooth and triangular. Mesopleuron smooth. Precoxal sulcus, deep and scrobiculate, running along the entire length of mesopleuron. Subalar sulcus deep and scrobiculate. Metanotum with a distinct median carina-like projection. Metapleuron entirely areolate-rugose. Propodeum uniformly areolate-rugose, with two longitudinal carinae joined basally and that immediately diverge forming an areola-like structure.

*Wings*: fore wing length 3.6 mm, length/width ratio 3.7; vein 1cu-a slightly postfurcal to vein 1M, thus vein (RS+M)b present (Fig. 18); veins 2RS/2M ratio 0.5.

*Legs*: fore tibia with a row of spines. Hind coxa transversally striate-rugose, with a small but distinct basoventral tubercle..

*Metasoma*: Basal sternal plate/length of first tergum 0.6. First metasomal tergite 2.5 times longer than apically wide (Fig. 19). Second median tergite longitudinally costate on basal three fourths, smooth on apical fourth. Suture between second and third median tergites sinuate laterally. Remaining terga smooth and polished. Ovipositor length 3.5 mm, 1.8 times longer than metasoma.

#### Variation.

Body length 3.4–4.3 mm. Temple/eye length ratio in dorsal view 0.4–0.5. Antenna with 26–28 flagellomeres. Prescutellar sulcus with four or five carinae. Fore wing length 3.5–3.6 mm, length/width ratio 3.7–3.8 times its maximum width. Ovipositor length 3.5–4.3 mm, 1.8-2.0 times longer than metasoma.

**Males.** Unknown.

#### Distribution.

Known only from the type locality in southern Cuba.

#### Biology.

Unknown.

#### Etymology.

This species is named after the Caribbean country where it occurs, Cuba.

#### Material examined.

Holotype (CNIN): Female, Cuba, Santiago, Gran Piedra Isabélica, 06-14/VII/1995, FIT, Cloud Forest, 1100m, S.B. Peck, DNA voucher number CNIN413, GenBank accession numbers JN870310 (COI), JN870491 (cyt *b*), KC822012 (EF-1alpha; not included in this work), KC822095 (*wingless*; not included in this work). Paratype (CNIN): one female, same data as holotype; DNA voucher number CNIN414, GenBank accession numbers JN870311 (COI), JN870492 (cyt *b*), JN870651 (*wingless*; not included in this work).

### Sergey tzeltal

Taxon classificationAnimaliaHymenopteraBraconidae

Martínez & Zaldívar-Riverón sp. n.

http://zoobank.org/7ECECEFC-2CB3-44C2-B544-D5A5FBB18FBF

Figs 20–23 , 24–29

#### Diagnosis.

This species is similar to *Sergey tzotzil*, but it can be distinguished from the latter species by the colour pattern of the white band on the female antenna. In *Sergey tzeltal*, the white band is either apical or subapical and is composed of at least two entire whitish flagellomeres, usually more, with at most three apical flagellomeres brown. In *Sergey tzotzil*, the white band is subapical and consists only of the lighter color on the articulation between the 19^th^ and 20^th^ flagellomeres, and with the five apical flagellomeres brown.

#### Description.

Body length 3.1 mm (Fig. 20), fore wing 2.7 mm; ovipositor sheaths 1.5mm. *Colour*: head uniformly brown, antenna brown, gradually darkening towards apex, except for a subapical white band composed of 2–4 flagellomeres (apical in Oaxaca population composed of 6-8 flagellomeres, see below) (Figs 24, 26). Mesosoma uniformly dark brown, except for a slightly lighter area on the median area of mesoscutum. Metasoma brown. Legs light brown, except fore and middle coxae, trochanters and trochantelli and hind trochantellus, which are pale yellow; hind coxa and apical three fourths of hind femur dark brown. Wings hyaline; pterostigma and veins brown. Ovipositor sheaths brown.

*Head*: 0.8 times as high as wide in anterior view (Fig. 21), and 0.7 times as long as wide in dorsal view (Fig. 22). Clypeus, face, frons and vertex smooth and polished; temple smooth. Eye 1.3 times higher than wide. Malar space height/eye height ratio 0.3. Temple/eye length ratio (dorsal view) 0.6. Antenna with 24 flagellomeres, first flagellomere about 4.0 times longer than wide, as long as second one.

*Mesosoma*: 2.0 times longer than wide (Fig. 22), 2.1 times longer than high (Fig. 23). Pronotal groove wide, deep and scrobiculate, pronotal carina distinct. Propleuron smooth. Mesoscutum slightly transverse, 0.7 times as long as wide. Mesoscutal lobes smooth, notauli deep and scrobiculate, obscured in an irregularly rugose median area before reaching the scuto-scutellar suture. Prescutellar sulcus with three distinct carinae. Scutellar disc smooth and triangular. Mesopleuron smooth. Precoxal sulcus deep and scrobiculate, running along basal two-thirds of mesopleuron. Subalar sulcus deep and rugose. Metanotum with a distinct median carina-like projection. Metapleuron entirely areolate-rugose. Propodeum with two divergent carinae running from median anterior edge delimiting two smooth dorsolateral areas; area beyond these carinae almost uniformly areolate-rugose.

*Wings*: fore wing (Fig. 28) length 3.0 mm, length/width ratio 3.85; vein 1cu-a postfurcal to vein 1M; veins 2RS/2M ratio 0.5.

*Legs*: fore tibia with a row of spines. Hind coxa transversally striate dorsally, smooth ventrally, with a distinct basoventral tubercule.

*Metasoma*: Basal sternal plate/length of first tergum 0.6. First metasomal tergite 2.2 times longer than apically wide (Fig. 29). Second median tergite longitudinally costate on basal three fourths, smooth apically. Suture between second and third median tergites slightly sinuate. Remaining tergites smooth and polished. Ovipositor length 1.4 mm, 0.9 times as long as metasoma.

#### Variation.

Body length 2.6–3.8 mm. Temple/eye length ratio in dorsal view 0.16–0.33. Antenna with 22-27 flagellomeres, white subapical band composed of two to four flagellomeres. In smaller specimens, rugose median area of mesoscutum reduced, though notauli never clearly distinguishable at posterior edge of mesoscutum, obscured among rugosities. Prescutellar sulcus sometimes with para-median carinae reduced, thus only the median carina is clearly distinguishable. Fore wing length 2.2–2.9 mm, length/width ratio 2.9–3.9 times its maximum width. Veins 2RS/2M ratio 0.5–0.55. Basal sternal plate 0.53–0.68 times length of first metasomal tergum. Ovipositor length 2.0–2.1 mm.

#### Males.

Body length 2.5–3.5 mm. Malar space 0.28. Temple/eye length ratio (dorsal view) 0.29–0.39. Flagellomeres 21–26 either entirely brown (Chiapas) (Fig. 27) or with two apical flagellomeres whitish (Oaxaca) (Fig. 25). Median apex of mesoscutum slightly coriaceous. Venter of mesosoma coriaceous to slightly coriaceous. Metapleuron coriaceous, coriaceous to slightly rugose distally. Fore wing 1cu-a vein slightly postfurcal to vein1M.2RS/2Mratio 0.47–0.51. Basal sternal plate 0.54 times length of first metasomal tergite.

#### Distribution.

This species is known from cloud forests located in the Reserva el Triunfo, Chiapas, and Santiago Comaltepec, Oaxaca, in southeast Mexico.

#### Biology.

Unknown.

#### Comments.

This species has a considerable variation in the antennal color pattern. We had originally grouped the specimens assigned to this taxon in two morphospecies, each represented by the specimens from Chiapas and Oaxaca, respectively. Females from Oaxaca have a distinct apical white band composed of 6–8 flagellomeres (Fig. 24), whereas in males this apical band is smaller (Fig. 25). On the other hand, most females from Chiapas have a white antennal band that is subapical and is only composed of 2-4 flagellomeres (Fig. 26). However, one female that could not be sequenced has an apical band similar to the specimens from Oaxaca. Other external morphological features (*e.g*. sculpture of propodeum and first metasomal tergite) also varied but we could not find any correlation with the geographical provenance of the specimens.

There was no concordance between the corrected COI distances and the geographic provenance and morphological variation for the above specimens. Some of the specimens from Oaxaca had lower COI distances with those from Chiapas than with the remaining specimens from the same locality (0.38–0.76 and 1.7–1.9%, respectively). This incongruence suggests that the existence of incomplete lineage sorting or hybridization between two recently diverged, sympatric species (see below). We have followed a conservative approach and consider the members of the populations from Chiapas and Oaxaca as a single species. One of the specimens from Oaxaca (DNA voucher number CNIN573) has considerably higher COI distances compared with the remaining conspecific specimens (3.6–4.7%). However, it is morphologically undistinguishable and we thus placed it within *Sergey tzeltal*.

#### Etymology.

The name of this species refers to the Tzeltal ethnic group, descendant from the Mayans that inhabits Los Altos, a mountain region located in central Chiapas

#### Material examined.

*Holotype* (CNIN): female, Mexico, Chiapas, Mpo. Albino Corzo, Reserva el Triunfo, 15°39.428N, 92°48.67W, YPT, 16/XI/2001, Kovarik col., DNA voucher number CNIN-711, GenBank accession numbers KC821997 (COI), KC822257 (cyt *b*), KC822078 (EF-1alpha; not included in this work), KC822122 (*wingless*; not included in this work). *Paratypes* (CNIN, MACN): one female, five males, same data as holotype, DNA voucher numbers CNIN712-15, 18, GenBank accession numbers KX074181-84, 87 (COI), KX074190-93 (cyt *b*), KX074195-97, 200 (28S); two females, same data as holotype except 15°39.447N, 92°48.40W, 17-20/XI/2001, DNA voucher numbers CNIN720-21, GenBank accession numbers KX074188-89 (COI); two females, five males, Mexico, Oaxaca, Mpio. Santiago Comaltepec, 17.62836 -96.4672; 6-8/VI/2009, YPT, 1495m, A. Zaldívar, H.Clebsch, DNA voucher numbers CNIN457-59, 461-64, GenBank accession numbers JN870332-34, 36-39 (COI), JN870515, 17-19 (cyt *b*), JN870673-74, 76-79 (*wingless*; not included in this work); one female, four males, Mexico, Oaxaca, Mpio. Santiago Comaltepec, 17.59056 -96.39902; 8/VI/2009, bosque mesófilo, 1998-2141m, A. Zaldívar, H.Clebsch, DNA voucher numbers CNIN468-71, GenBank accession numbers JN870343-46 (COI), JN870524 (cyt *b*), JN870681-84 (*wingless*; not included in this work); one male, Mexico, Oaxaca, Mpio. Santiago Comaltepec, La Esperanza, 17.62661 -96.36950; 8/VI/2009, cloud forest, 1600m, A. López, DNA voucher number CNIN479, GenBank accession number JN870352 (COI), KX074194 (cyt *b*), JN870689 (*wingless*; not included in this work); two females, five males, Mexico, Oaxaca, Mpio. Santiago Comaltepec, 17.62334 -96.34669; 6/V/2009, bosque mesófilo, 1460m, A. Zaldívar, DNA voucher numbers CNIN379-80, 446-48, 452-53, GenBank accession number JN870324-26, 295-96 (COI), JN870509-11, 466 (cyt *b*), JN870630-31, 64-65 (*wingless*; not included in this work), KC822056-57 (EF-1alpha; not included in this work).

### Sergey tzotzil

Taxon classificationAnimaliaHymenopteraBraconidae

Martínez & Zaldívar-Riverón sp. n.

http://zoobank.org/7910978A-AFB7-41D2-B064-BBE99700B5E8

Figs 30–35 , 36–37

#### Diagnosis.

See diagnosis of *Sergey tzeltal*.

#### Description.

Body length 3.7mm (Fig. 30), fore wing 3.2mm; ovipositor sheaths 2.3mm. *Colour*: head uniformly brown, antenna brown, gradually darkening towards apex, except for a light band composed of most of the 19^th^ and the basal half of the 20^th^ flagellomeres (Fig. 34). Mesosoma uniformly dark brown, except for a slightly lighter area on median area of mesoscutum. Metasoma brown. Legs light brown, except fore and middle coxae, trochanters and trochantelli and hind trochantellus which are pale yellow; hind coxa and apical three fourths of hind femur dark brown. Wings hyaline; pterostigma and veins brown. Ovipositor sheaths brown.

*Head*: in anterior view 0.9 times as high as wide (Fig. 31), and 0.6 times as long as wide in dorsal view (Fig. 32). Clypeus, face, frons and vertex smooth and shining; temple smooth (Fig. 33). Eye 1.3 times higher than wide. Malar space height/eye height ratio 0.4. Temple/eye length ratio (dorsal view) 0.6. Antenna with 25 flagellomeres, first flagellomere five times longer than wide and about as long as the second one.

**Figures 36–37.**
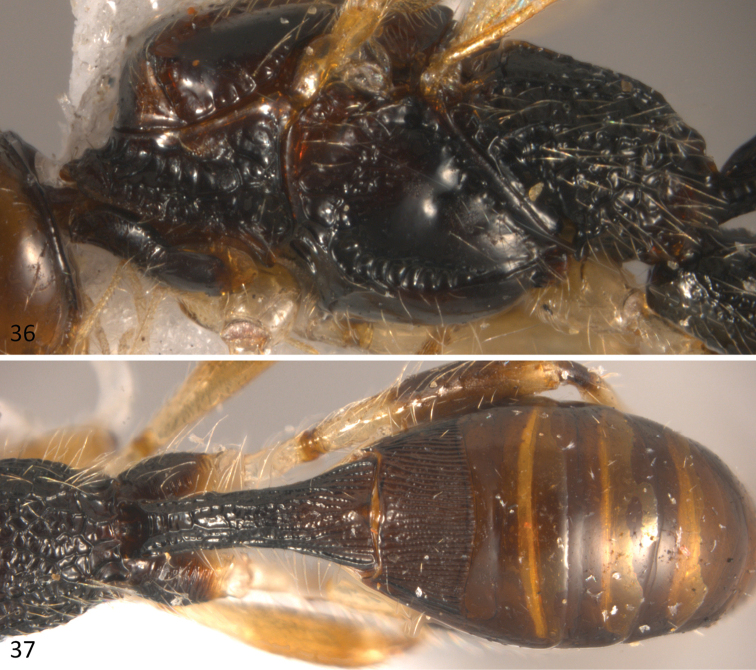
*Sergey tzotzil* sp. n.; **36** mesosoma in lateral view **37** metasoma in dorsal view.

*Mesosoma*: 2.1 times longer than wide (Fig. 35) and 2.0 times longer than high (Fig. 36). Pronotal groove wide, deep, and scrobiculate, pronotal carina distinct. Propleuron smooth. Mesoscutum slightly transverse, 0.7 times as long as wide. Mesoscutal lobes smooth, notauli deep and scrobiculate, obscured in an irregularly rugose median area before reaching the scuto-scutellar suture. Prescutellar sulcus with three distinct carinae. Scutellar disc smooth and triangular. Mesopleuron smooth. Precoxal sulcus, deep and scrobiculate, running along basal two-thirds of mesopleuron. Subalar sulcus deep and scrobiculate-rugose. Metanotum with a distinct median projection. Metapleuron entirely areolate rugose. Propodeum with two divergent carinae running fron median anterior edge delimiting two dorsolateral areas, these areas are mostly smooth, but turn rugose areolate near carinae; beyond these carinae the propodeum almost uniformly areolate-rugose.

*Wings*: Fore wing length 2.9 mm, length/width ratio 3.3; vein 1cu-a slightly postfurcal to vein 1M; veins 2RS/2M ratio 0.5.

*Legs*: Fore tibia with a row of spines. Hind coxa transversally striate dorsally, smooth ventrally, with a distinct basoventral tubercle.

*Metasoma*: Basal sternal plate/length of first tergum 0.6. First metasomal tergite 2.1 times longer than apically wide (Fig. 37). Second median tergite longitudinally costate on basal three fourths, smooth apically. Suture between second and third median tergites almost straight. Remaining terga smooth and polished. Ovipositor length 2.4 mm, 1.1 times as long as metasoma.

**Males.** Similar to female, slightly smaller and with antenna uniformly brown.

#### Distribution.

Known only from the type locality in El Triunfo, Chiapas, Mexico.

#### Biology.

Unknown.

#### Comments.

This species and *Sergey tzeltal* were collected in the same locality in Chiapas.

#### Etymology.

The name of this species refers to the Tzotzil ethnic group, descendant from the Mayans, who inhabits the Altos, a mountain region located in central Chiapas.

#### Material examined.

*Holotype* (CNIN): female, Mexico, Chiapas, Mpo. Albino Corzo, Reserva el Triunfo, 15°39.428N, 92°48.67W, YPT, 16/XI/2001, Kovarik col., DNA voucher number CNIN717, GenBank accession nos. KC821999 (COI), KC822259 (cyt *b*), KX074199 (28S), KC822124 (wingless, not included in this work), KC822080 (EF-1alpha; not included in this work). Paratype (CNIN): one male, same data as holotype; DNA voucher number, CNIN716, DNA voucher nos. KC821998 (COI), KC822258 (cyt *b*), KX074198 (28S), KC822123 (*wingless*, not included in this work), KC822079 (EF-1alpha; not included in this work).

#### Gene genealogies.

Intraspecific corrected genetic divergences varied from 0 to 2.1 (excluding CNIN573), 0.27 to 3.18 and 0 to 0.33% for COI, cyt *b* and 28S, respectively. Interspecific distances within *Sergey* on the other hand ranged from 7.99 to 15.28, 12.64 to 13.6 and 0.17 to 0.5% for COI, cyt *b* and 28S, respectively.

**Figure 38.**
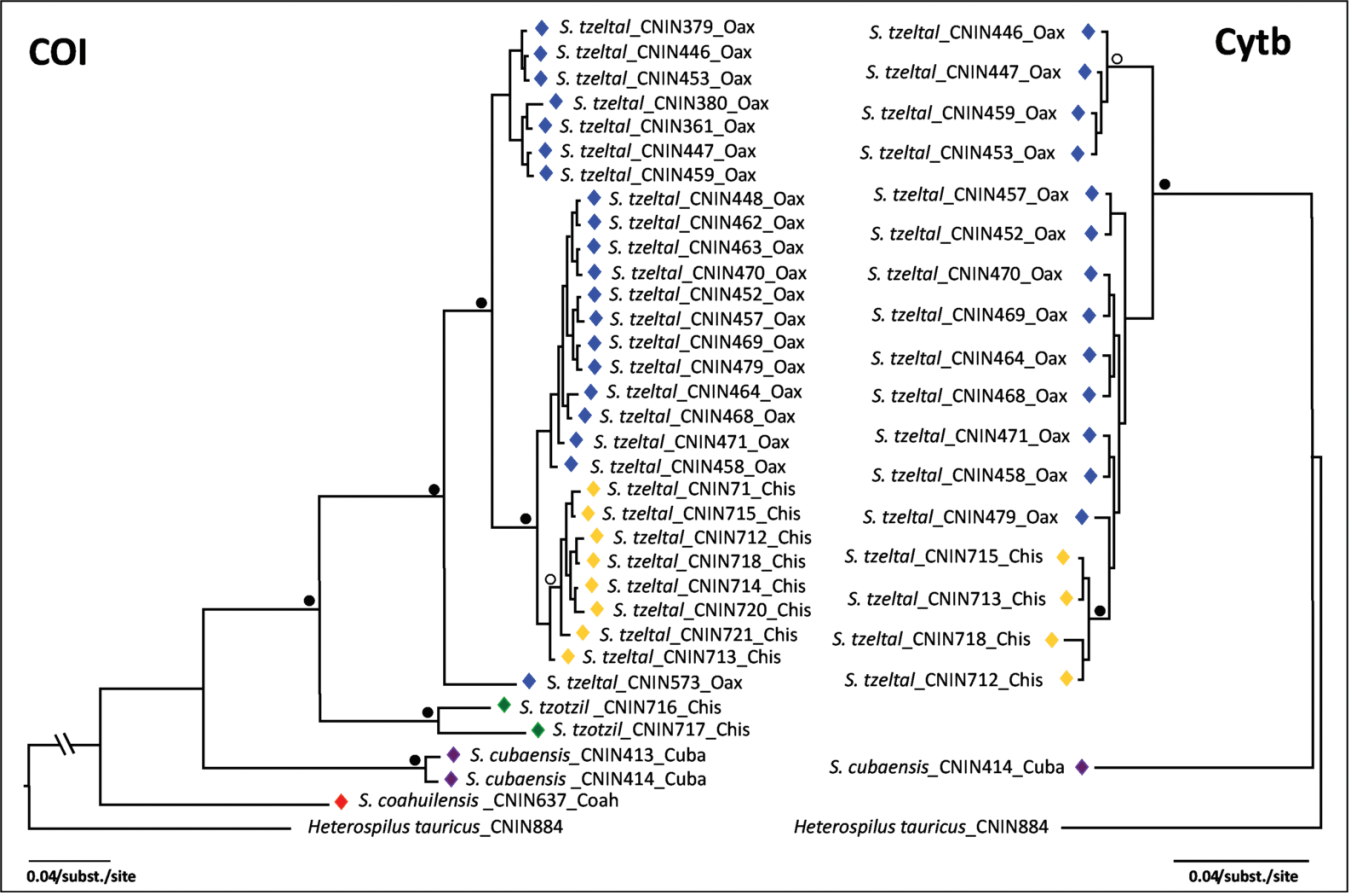
Bayesian phylograms showing the relationships recovered by the separate COI and cyt *b* analyses. Black circles near branches indicate clades supported by posterior probabilities ≥ 95%; hollow circles near branches indicate clades supported by posterior probabilities ≥ 90 and ≤ 94%.

The Bayesian phylograms derived from the separate COI and cyt *b* analyses are included in the Figure 38. The COI bayesian phylogram significantly supported the monophyly of the three described species. *Sergey coahuilensis* was recovered as sister to *Heterospilus* but without statistical support (PP = 0.5). A clade with the remaining species of *Sergey* (PP = 0.5) recovered *Sergey cubaensis* from Cuba (PP = 0.5) as sister to a *Sergey tzotzil* (Chiapas) + *Sergey tzeltal* (Chiapas and Oaxaca) clade (PP = 1.0). Within *Sergey tzeltal*, there were three non-significantly supported, subclades, two of which were composed of specimens from Comaltepec, Oaxaca, but with one of them being more closely related to the subclade containing the specimens from El Triunfo, Chiapas.

The bayesian phylogram derived from the cyt *b* sequences yielded similar relationships with the COI topology. Again, some of the specimens of *Sergey tzeltal* from Comaltepec, Oaxaca were more closely related to the ones from El Triunfo, Chiapas (PP = 0.6) than with the remaining specimens from the same locality. The 28S tree was largely unresolved (phylogram not shown), with the sequenced specimens *Sergey tzeltal* and *Sergey tzotzil* grouped together (PP = 1.0). The reconstructed mt gene genealogies, together with the geographic provenance and morphological variation found in the specimens of *Sergey tzeltal* from Oaxaca and Chiapas suggests that this taxon could consist of two sympatric, recently derived lineages in which there is incomplete lineage sorting or hybridization. Further morphological and genetic studies will help to confirm the taxonomic status of the populations of *Sergey tzeltal* from the latter two Mexican regions.

## Supplementary Material

XML Treatment for Sergey

XML Treatment for Sergey coahuilensis

XML Treatment for Sergey cubaensis

XML Treatment for Sergey tzeltal

XML Treatment for Sergey tzotzil
